# Research on GNSS/MEMS IMU Array Fusion Localization Method Based on Improved Grey Prediction Model

**DOI:** 10.3390/mi16091040

**Published:** 2025-09-11

**Authors:** Yihao Chen, Jieyu Liu, Weiwei Qin, Can Li

**Affiliations:** 1 College of Missile Engineering, Rocket Force University of Engineering, Xi’an 710025, China; cyhao173@163.com (Y.C.); 15339020629@163.com (C.L.); 2 School of Nuclear Engineering, Rocket Force University of Engineering, Xi’an 710025, China; qww_1982@163.com

**Keywords:** vehicle navigation, GNSS denial, MEMS IMU arrays, grey prediction model, adaptive fusion

## Abstract

To address the issue of decreased positioning accuracy caused by interference or blockage of GNSS signals in vehicle navigation systems, this paper proposes a GNSS/MEMS IMU array fusion localization method based on an improved grey prediction model. First, a multi-feature fusion GNSS confidence evaluation algorithm is designed to assess the reliability of GNSS data in real time using indicators such as signal strength, satellite visibility, and solution consistency; second, to overcome the limitations of traditional grey prediction models in processing vehicle complex motion data, two key improvements are proposed: (1) a dynamic background value optimization method based on vehicle motion characteristics, which dynamically adjusts the weight coefficients in the background value construction according to vehicle speed, acceleration, and road curvature, enhancing the model’s sensitivity to changes in vehicle motion state; (2) a residual sequence compensation mechanism, which analyzes the variation patterns of historical residual sequences to accurately correct the prediction results, significantly improving the model’s prediction accuracy in nonlinear motion scenarios; finally, an adaptive fusion framework under normal and denied GNSS conditions is constructed, which directly fuses data when GNSS is reliable, and uses the improved grey model prediction results as virtual measurements for fusion during signal denial. Simulation and vehicle experiments verify that: compared to the traditional GM(1,1) model, the proposed method improves prediction accuracy by 31%, 52%, and 45% in straight, turning, and acceleration scenarios, respectively; in a 30-s GNSS denial scenario, the accuracy is improved by over 79% compared to pure INS methods.

## 1. Introduction

Microelectromechanical system-inertial navigation system/global navigation satellite system (MEMS-INS/GNSS) is a classic integrated navigation method, widely used in vehicle, drone, and other navigation fields [1]. MEMS-INS has advantages such as low cost, small size, and low power consumption, and is widely used in vehicle-mounted navigation systems [2]. However, MEMS-INS has inherent defects such as high noise and severe zero-bias drift. During GNSS signal rejection, the error of pure inertial navigation will rapidly diverge over time [3]. At the same time, in complex environments such as urban canyons, tunnels, and under overpasses [4], signals are easily affected by building obstructions, multipath effects, and electromagnetic interference, leading to deterioration or complete interruption of signal quality, which seriously restricts the positioning performance and reliability of vehicle-mounted navigation systems [5]. These issues urgently need technological innovation to solve, in order to meet the high-precision navigation and autonomous driving safety requirements [6].

To address the performance degradation of GNSS/MEMS-INS integrated navigation in signal jamming environments, current research mainly develops along three technical directions [7]. Traditional filtering fusion methods, as classic techniques in the field of integrated navigation, include Extended Kalman Filter (EKF) [8] and Unscented Kalman Filter (UKF) [9], which can effectively fuse multi-source information under normal GNSS signal conditions to achieve high-precision positioning. Wen et al. [10] demonstrated through a comparative study of factor graph optimization method [11] and EKF that both filtering methods perform well in open environments. Similar work by Jia et al. [12] on GNSS/INS/Visual integration further validated the effectiveness of factor graph approaches. However, the fundamental limitation of these methods is that when GNSS signals are denied [13], the system can only rely on INS for trajectory estimation. Due to the inherent error characteristics of MEMS devices, the positioning accuracy rapidly declines over time, making it difficult to maintain long-term high-precision navigation. Machine learning prediction methods have shown great potential in GNSS/INS integrated navigation in recent years [14]. Neural networks and deep learning techniques are widely used to predict position information during GNSS signal denial. Dai et al. [15] proposed an INS/GNSS integrated navigation method based on recurrent neural networks, achieving good prediction results in GNSS jamming environments. Song et al. [16] further improved LSTM-assisted vehicle-mounted GNSS/SINS navigation, demonstrating enhanced positioning accuracy. Alaeiyan et al. [17] used an incremental regularization LSTM learning approach to further improve navigation accuracy during GNSS interruptions. Xu et al. [18] addressed some of these challenges through motion-constrained GNSS/INS integration using BP neural networks. However, machine learning methods face significant engineering implementation challenges: they require large amounts of training data specific to particular scenarios, involve high computational complexity for model training and inference, and have limited generalization ability under complex vehicle motion conditions, often showing significant performance drops outside the training scenarios [19]. The grey system prediction method [20], due to its good adaptability to small samples and uncertain systems, has gradually attracted attention in navigation [21]. Zheng et al. [22] applied an improved grey model to real-time GPS satellite clock difference prediction, verifying its effectiveness. In the field of integrated navigation, Li et al. [23] proposed a grey prediction model-based integrated navigation method, which can replace faulty data with predicted data during brief GPS failures to enhance system anti-interference capability. However, its accuracy significantly declines when data fluctuations are large [24]. Zhang et al. [25] proposed the GP-MLP hybrid algorithm, which improves prediction accuracy to some extent, but its background value construction still uses traditional fixed-weight methods, failing to dynamically optimize based on vehicle motion characteristics, and lacking an effective residual compensation mechanism. Comprehensive analysis shows that each existing method has its advantages but also key limitations: traditional filtering methods are theoretically mature and simple to implement but lack effective prediction capabilities during signal denial; machine learning methods offer higher prediction accuracy but require large data, involve high computational complexity, and have poor practical engineering applicability; grey system methods are simple to compute and require less data but cannot effectively handle the nonlinear features and dynamic changes of vehicle motion [26]. The fundamental reason for these technical limitations is that current methods do not fully consider the dynamic characteristics and kinematic constraints of vehicle motion, highlighting the urgent need for theoretical innovation and algorithm optimization tailored to specific onboard navigation scenarios.

For this reason, this paper proposes a GNSS/MEMS-INS integrated positioning method based on an improved grey prediction model, with the main contributions as follows:

(1) Multi-feature fusion GNSS confidence assessment algorithm: Designed a GNSS data quality evaluation system that integrates multiple indicators such as signal strength, satellite visibility, geometric accuracy factors, and solution consistency, enabling real-time dynamic assessment of GNSS data reliability and providing a reliable basis for subsequent adaptive fusion strategies.
(2) Dynamic background value optimization method based on vehicle motion characteristics: Breaks through the limitations of the traditional grey model’s fixed background value construction, proposing an innovative method to dynamically adjust the weighting coefficients in the background value construction based on real-time vehicle speed, acceleration, and road curvature, significantly improving the model’s sensitivity and adaptability to changes in vehicle motion states.
(3) Residual sequence compensation mechanism: Addressing the nonlinear trajectory characteristics of vehicles, established a prediction error correction model based on historical residual sequence analysis, exploiting residual variation patterns to achieve precise compensation of prediction results, effectively enhancing the model’s prediction accuracy in complex motion scenarios.
(4) Adaptive fusion framework: Constructed an intelligent switching fusion architecture for GNSS normal/rejection scenarios, achieving optimal fusion of multi-source information when GNSS signals are reliable, and seamlessly switching to a virtual measurement fusion mode based on the improved grey model during signal rejection, ensuring continuous and stable positioning performance.

## 2. Confidence Algorithm and Improved Grey Prediction Model

### 2.1. GNSS Confidence Level Evaluation Algorithm

#### 2.1.1. GNSS Signal Status Analysis and Classification

In vehicle navigation applications, the reception quality of GNSS signals is affected by various factors, resulting in complex variations in signal status. Based on signal characteristics and reliability levels, GNSS signal status can be divided into three main categories [27]. Normal signal status refers to high-quality satellite signals received in open environments, characterized by a carrier-to-noise ratio $C/N_{0}$ greater than 35 dB-Hz, with at least four visible satellites, a geometric dilution of precision (GDOP) less than 6, and pseudorange measurement residuals within 5 m. Under this condition, the GNSS system can provide stable and reliable positioning services, meeting the accuracy requirements of vehicle navigation.

The degraded signal quality state is mainly caused by environmental factors and includes three typical scenarios. Weak signal environments often occur at building edges, underground entrances, and similar 25~35 dB-Hz locations, where the carrier-to-noise ratio drops to within range, with severe signal power attenuation but still maintaining basic signal tracking. Obstructed environments refer to tunnels, under elevated bridges, dense building clusters, and similar scenarios, characterized primarily by insufficient visible satellites, a significant increase in GDOP, and a marked decrease in positioning accuracy. Multipath interference environments involve signals reflected by buildings or the ground before reaching the receiver, leading to increased pseudorange measurement errors, frequent cycle slips in carrier phase, and position solutions oscillating around the true position.

The abnormal signal state refers to a signal condition affected by intentional attacks or severe interference, posing a direct threat to the security of the navigation system. Spoofing signals are artificially generated false GNSS signals, characterized by features such as abnormally high carrier-to-noise ratio (usually exceeding 50 dB-Hz), multiple satellite signals appearing simultaneously within a short period, abnormal jumps in positioning results, and discontinuities in timestamps. Interference signals mainly manifest as broadband noise interference, causing a rapid decline in signal quality across all frequency bands and abnormal responses in automatic gain control of the receiver. Severe multipath effects, although not caused by intentional attacks, can lead to drastic fluctuations in signal power, frequent jumps in pseudorange measurements, and significant discontinuities in carrier phase, all of which can compromise positioning reliability.

#### 2.1.2. Multidimensional Confidence Level Evaluation Model Design

To address the complexity and diversity of GNSS signal states, this paper proposes a confidence level evaluation model based on the fusion of multiple indicators. The model decomposes the signal trustworthiness assessment into five interrelated yet relatively independent evaluation dimensions, and obtains a comprehensive confidence score through weighted fusion. The mathematical expression for the comprehensive confidence is:

(1) $$C_{\mathrm{GNSS}}{\;=\;w}_{1}\times S_{1}+w_{2}\times S_{2}+w_{3}\times S_{3}+w_{4}\times S_{4}+w_{5}\times S_{5}$$

where C_GNSS_ is the overall confidence score, the value range is [0, 1]. w_i_ is the corresponding weight coefficient. The weight coefficients are determined through the Analytic Hierarchy Process and extensive experimental data analysis, and are respectively based on signal strength w_1_ = 0.30, geometric configuration w_2_ = 0.25, solution consistency w_3_ = 0.20, temporal continuity w_4_ = 0.15, and anomaly detection w_5_ = 0.10.

This weighting scheme emphasizes the fundamental roles of signal strength and geometric configuration while also fully considering the importance of solution consistency, temporal continuity, and anomaly detection in security assessment.

The assessment of signal strength mainly relies on the Carrier-to-Noise Density (*C*/*N*0) metric, which directly reflects the ratio of received signal power to noise power. The calculation formula for the carrier-to-noise ratio score is:

(2) $$\left\{\begin{array}{cc} S_{1}=0 & C/NO<20\text{ dB-Hz}\\ S_{1}=(C/NO-20)/25 & 20\leq C/NO\leq 45\text{ dB-Hz}\\ S_{1}=1 & C/NO>45\text{ dB-Hz} \end{array}\right.$$

When the carrier-to-noise ratio is below 20 dB-Hz, the signal quality is extremely poor, and the confidence level is 0; when the carrier-to-noise ratio is within the range of 20 dB-Hz to 40 dB-Hz, a linear mapping function is used; when the carrier-to-noise ratio exceeds 45 dB-Hz, although the signal strength is high, caution should be taken for possible spoofing attacks. In this case, the confidence level is set to 1 but will be further evaluated in the anomaly detection dimension.

The geometric configuration assessment considers both the number of visible satellites and the geometric dilution of precision (GDOP), with the calculation formula as follows:

(3) $$S_{2}=\alpha \cdot S_{sat}+(1-\alpha )\cdot S_{GDOP}$$

where α=0.6 is the weight coefficient. $S_{sat}$ is the satellite count score, and $S_{GDOP}$ is the GDOP score. The satellite count score is calculated as:

(4) $$\left\{\begin{array}{cc} S_{sat}=0 & N_{sat}<4\\ S_{sat}=(N_{sat}-4)/8 & 4\leq N_{sat}\leq 12\\ S_{sat}=1 & N_{sat}>12 \end{array}\right.$$

The GDOP score is calculated as:

(5) $$\left\{\begin{array}{cc} S_{GDOP}=0 & GDOP>10\\ S_{GDOP}=(10-GDOP)/8 & 2\leq GDOP\leq 10\\ S_{GDOP}=1 & GDOP<2 \end{array}\right.$$

The solution consistency evaluation assesses signal reliability by analyzing the differences in position results obtained through three methods: least squares, weighted least squares, and carrier phase smoothing pseudorange solutions. The calculation formula is:

(6) $$S_{3}=\exp(-{\sigma_{pos}}^{2}/2{\sigma_{0}}^{2})$$

where $\sigma_{pos}$ is the standard deviation of the position results from the three solution methods. $\sigma_{0}=5\;m$ is the reference threshold. When the solution results are consistent, the standard deviation is small, and the confidence level approaches 1; when the differences are large, it indicates signal anomalies, and the confidence decreases.

The temporal continuity assessment mainly detects the smoothness of position solutions over time by analyzing the positional changes between consecutive epochs to identify abnormal jumps. The calculation formula is:

(7) $$S_{4}=\exp(-{\left({\Delta P}_{k}\right)}^{2}/(2\sigma_{v}^{2}))$$

where ${\Delta P}_{k}$ is the positional change between the current epoch and the previous epoch. Considering vehicle motion constraints, $\sigma_{v}$ is dynamically adjusted based on the current vehicle speed and time interval:

(8) $$\sigma_{v}=v\times \Delta t+0.5\times a_{max}\times {\left(\Delta t\right)}^{2}$$

where v is the current vehicle speed. Δt is the sampling interval, and $a_{max}=8\;m/s^{2}$ is the maximum possible acceleration.

The anomaly detection dimension is specifically designed to detect spoofing attacks and severe interference, using a combination of multiple indicators for joint judgment. The calculation formula is:

(9) $$S_{5}=S_{\mathrm{spoof}}\times S_{\mathrm{jam}}\times S_{\mathrm{multipath}}$$

Spoofing detection is based on three features: carrier-to-noise ratio anomalies, power correlation, and clock jumps: when C/NO > 50 dB-Hz, $F_{1}$ = 1; when multiple satellites’ power levels change anomalously simultaneously, $F_{2}$ = 1; when the receiver clock exhibits abnormal jumps, $F_{3}$ = 1; otherwise, the feature values are 0. The spoofing detection score is:

(10) $$S_{spoof}=1-0.8\times (F_{1}+F_{2}+F_{3})/3$$

Interference detection is based on abnormal responses of automatic gain control (AGC), calculated as:

(11) $$S_{\mathrm{jam}}=1-\tanh(|\mathrm{AGC}-{\mathrm{AGC}}_{\mathrm{ref}}{|/\mathrm{AGC}}_{\mathrm{th}})$$

where ${\mathrm{AGC}}_{\mathrm{ref}}$ is the reference AGC value. ${\mathrm{AGC}}_{\mathrm{th}}$ is the detection threshold. Multipath detection is evaluated through the continuity of carrier phase, with the calculation formula as:

(12) $$S_{\mathrm{multipath}}=\exp\left(-N_{\mathrm{cycle}-\mathrm{slip}}/N_{\mathrm{total}}\right)$$

where $N_{\mathrm{cycle}-\mathrm{slip}}$ is the number of cycle slips detected, and $N_{\mathrm{total}}$ is the total number of observation epochs.

#### 2.1.3. Comprehensive Evaluation

The real-time calculation of the integrated confidence level adopts a sliding window smoothing mechanism to reduce the impact of instantaneous interference, with the calculation formula as:

(13) $$C_{\mathrm{GNSS}}(k)=\lambda \times C_{\mathrm{GNSS}}(k)+(1-\lambda )\times C_{\mathrm{GNSS}}(k-1)$$

where λ=0.3 is the smoothing coefficient, and k is the current epoch. The final confidence judgment rule is: when $C_{\mathrm{GNSS}}>0.8$, it indicates high confidence, and GNSS data can be directly used for fusion; when $0.5\leq C_{\mathrm{GNSS}}\leq 0.8$, it indicates medium confidence, and weighted fusion is applied; when $C_{\mathrm{GNSS}}<0.5$, it indicates low confidence, and a grey prediction model is activated.

This confidence evaluation algorithm uses real-time online computation with a time complexity of 0(n), where n is the number of visible satellites, fully meeting the real-time requirements of onboard navigation systems.

### 2.2. Improved Grey Prediction Model

Based on the GNSS confidence evaluation results, when the system detects a decline in signal quality ($C_{\mathrm{GNSS}}<0.6$), it is necessary to activate the grey prediction model to generate virtual GNSS measurements. However, traditional GM(1,1) models have limitations in onboard navigation applications. This paper improves the grey prediction model from two aspects: dynamic adjustment optimization methods and residual sequence compensation mechanisms.

#### 2.2.1. Traditional Grey Prediction Model

The model is the core of grey prediction theory, suitable for small sample prediction problems. The modeling process includes: performing an accumulated generation on the original sequence $X^{\left(0\right)}$ to obtain $X^{\left(1\right)}$, establishing the grey differential equation $\frac{dx^{\left(1\right)}}{dt}+ax^{\left(1\right)}=b$, estimating parameters using the least squares method, and finally deriving the prediction formula:

(14) $$\frac{dx^{\left(1\right)}}{dt}+ax^{\left(1\right)}=b$$

Traditional GM(1,1) models have advantages such as low data requirements, simple computation, and strong noise resistance. However, in vehicle trajectory prediction, there are obvious limitations: first, the background value construction is fixed, using fixed weights $z^{\left(1\right)}(k)=0.5[x^{\left(1\right)}(k-1)+x^{\left(1\right)}(k)]$, which cannot adapt to changes in vehicle motion states; second, it ignores residual information and does not fully utilize valuable information from prediction errors; third, based on linear assumptions, it is difficult to handle nonlinear vehicle movements such as turning, acceleration, and deceleration.

These limitations lead to insufficient prediction accuracy of traditional models in complex vehicle motion scenarios, necessitating targeted improvements based on vehicle motion characteristics.

#### 2.2.2. Dynamic Adjustment Optimization Method

The traditional model constructs the background value using a simple arithmetic mean of adjacent data, i.e.,:

(15) $$z^{\left(1\right)}(k)=\frac{1}{2}\left[x^{\left(1\right)}(k)+x^{\left(1\right)}(k-1)\right]$$

where $z^{\left(1\right)}(k)$ is the first-order accumulated generating sequence.

This fixed-weight background value construction method ignores the dynamic changes in vehicle motion state, especially in complex scenarios such as acceleration, deceleration, and turning, making it unable to accurately reflect the trend of position data. To address this issue, this paper proposes a dynamic background value optimization method based on vehicle motion characteristics. This method dynamically adjusts the weight coefficients in the background value construction according to the real-time motion state of the vehicle, enabling the model to better adapt to vehicle movement patterns.

First, features of the vehicle’s motion state are extracted based on IMU array data. The vehicle’s motion state vector is defined as:

(16) $$S(k)={\left[v(k),a(k),w(k),\kappa (k)\right]}^{T}$$

where v(k) is the vehicle speed, a(k) is the acceleration, w(k) is the angular velocity, κ(k) is the path curvature. Speed and acceleration are obtained by integrating the accelerometer data from the IMU array:

(17) $$v(k)=v(k-1)+a_{imu}(k)\cdot \Delta t$$

(18) $$a(k)=\frac{a_{imu}(k)-a_{imu}(k-1)}{\Delta t}$$

The path curvature is calculated based on the rate of change of the vehicle’s heading angle:

(19) $$\kappa (k)=\frac{\Delta \psi (k)}{v(k)\cdot \Delta t}$$

where Δψ(k) is the change in heading angle.

Based on extracted motion state features, a dynamic weight coefficient calculation method is designed. The dynamic construction formula of the background value is:

(20) $$z^{\left(1\right)}(k)=\alpha (k)\cdot x^{\left(1\right)}(k)+\left[1-\alpha (k)\right]\cdot x^{\left(1\right)}(k-1)$$

where α(k) is the dynamic weight coefficient, and its calculation formula is:

(21) $$\alpha (k)=\alpha_{0}+\beta_{v}\cdot f_{v}(k)+\beta_{a}\cdot f_{a}(k)+\beta_{\kappa }\cdot f_{\kappa }(k)$$

where $\alpha_{0}=0.5$ is the baseline weight, $\beta_{v}$, $\beta_{a}$, $\beta_{\kappa }$ is the adjustment coefficient, and $f_{v}(k)$, $f_{a}(k)$, $f_{\kappa }(k)$ are the normalized functions of speed, acceleration, and curvature respectively.

The speed normalization function is defined as:

(22) $$f_{v}(k)=\frac{v(k)-v_{\min}}{v_{\max}-v_{\min}}-0.5$$

where $v_{\min}$ is the minimum velocity value in the historical observation data, $v_{\max}$ is the maximum velocity value in the historical observation data, obtained through statistics of velocity data from the first N epochs before prediction.

The acceleration normalization function considers the absolute value of acceleration:

(23) $$f_{a}(k)=\frac{\left|a(k)\right|}{a_{\max}}\cdot sign(a(k))$$

where the sign(⋅) function is used to preserve the directional information of acceleration, distinguishing between acceleration and deceleration; $a_{\max}$ is the maximum acceleration in the historical observation data.

The curvature normalization function is defined as:

(24) $$f_{\kappa }(k)=\tanh(\kappa (k)/\kappa_{ref})$$

where tanh(⋅) is the hyperbolic tangent function, which implements soft saturation characteristics to avoid numerical instability caused by extreme curvature; $\kappa_{ref}$ is the reference curvature.

To ensure the stability of the model, constraints are imposed on the dynamic weight coefficient:

(25) $$\alpha (k)\in \left[0.2,0.8\right]$$

When the vehicle is in uniform straight-line motion, ($\left|a(k)\right|<a_{th}\wedge \left|\kappa (k)\right|<\kappa_{th}$), the weight coefficient tends toward 0.5, at which point the model degenerates into the traditional GM(1,1) model; when the vehicle is accelerating or turning, the weight coefficient adjusts dynamically to enhance the model’s sensitivity to current data.

#### 2.2.3. Residual Sequence Compensation Mechanism

The traditional GM(1,1) model often has insufficient prediction accuracy when dealing with nonlinear changes in vehicle position data. The residual sequence contains rich system error information and nonlinear features; properly utilizing this information can significantly improve the prediction accuracy of the model.

Let the predicted value of the original GM(1,1) model be ${\hat{x}}^{\left(0\right)}(k)$, and the actual observed value be ${\hat{x}}^{\left(0\right)}(k)$. Then, the residual sequence is defined as:

(26) $$e(k)=x^{\left(0\right)}(k)-{\hat{x}}^{\left(0\right)}(k)$$

Statistical analysis of the residual sequence reveals the following characteristics: First, it exhibits periodic characteristics, where the residual sequence tends to show certain periodic variations during vehicle operation due to the relative stability of road conditions and driving habits; second, it demonstrates trending characteristics, where the residual sequence shows obvious trends under specific motion patterns (such as continuous acceleration and turning); additionally, it contains stochastic characteristics, where the residual sequence includes random components influenced by factors such as road bumps and sensor noise.

According to different vehicle motion states, residual compensation is divided into three modes:

Linear motion mode: When in this mode, residuals are mainly caused by system bias, using a first-order autoregressive model:

(27) $$\hat{e}(k+1)=\phi_{1}\cdot e(k)+\epsilon_{1}(k)$$

Turning motion mode: When in this mode, residuals are related to curvature, using a curvature compensation model:

(28) $$\hat{e}(k+1)=e(k)+\delta \cdot \kappa (k)\cdot v(k)\cdot \Delta t$$

where δ is the curvature compensation coefficient.

Accelerated motion mode: When in this mode, residuals exhibit trend-like changes, using a trend extrapolation model:

(29) $$\hat{e}(k+1)=e(k)+\Delta e_{trend}(k)$$

where $\Delta e_{trend}(k)=\gamma \cdot a(k)\cdot \Delta t$; γ is the trend coefficient.

To ensure the accuracy of motion pattern discrimination, based on statistical analysis research from field testing, the acceleration threshold $a_{th}=0.4\;g$ and curvature threshold $\kappa_{th}=0.02\;m^{-1}$ are set.

To fully utilize residual features under various motion modes, a multimodal fusion residual prediction method is designed:

(30) $$\hat{e}(k+1)=w_{1}(k)\cdot {\hat{e}}_{1}(k+1)+w_{2}(k)\cdot {\hat{e}}_{2}(k+1)+w_{3}(k)\cdot {\hat{e}}_{3}(k+1)$$

where ${\hat{e}}_{1}$, ${\hat{e}}_{2}$, ${\hat{e}}_{3}$ represents the residual prediction values in the linear, acceleration, and turning modes respectively, and the weight coefficient calculation formula is:

(31) $$w_{1}(k)=\frac{\exp(-|a(k)|/a_{th}-|\kappa (k)|/\kappa_{th})}{\sum_{i=1}^{3}\exp(-s_{i}(k))}$$

(32) $$w_{2}(k)=\frac{\exp(|a(k)|/a_{th})}{\sum_{i=1}^{3}\exp(-s_{i}(k))}$$

(33) $$w_{3}(k)=\frac{\exp(|\kappa (k)|/\kappa_{th})}{\sum_{i=1}^{3}\exp(-s_{i}(k))}$$

The final predicted result after fused residual compensation is:

(34) $$x_{final}^{\left(0\right)}(k+1)={\hat{x}}^{\left(0\right)}(k+1)+\hat{e}(k+1)$$

To prevent excessive correction of residual compensation, a compensation limit condition is set:

(35) $$\left|\hat{e}(k+1)\right|\leq \sigma_{max}\cdot \sigma_{e}$$

where $\sigma_{e}$ is the standard deviation of the residual sequence, $\sigma_{max}$ is the limiting factor, usually taking values between 2 and 3.

**Figure 1:**
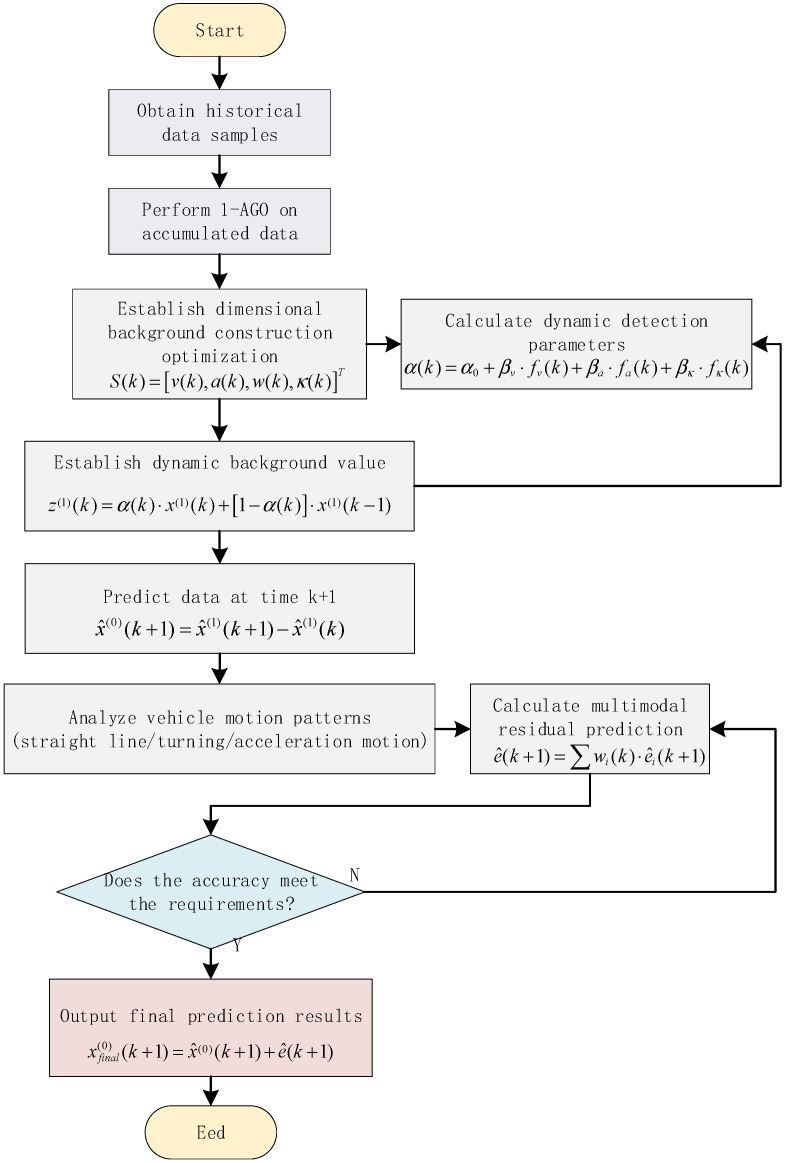
Flowchart of GNSS Virtual Observable Prediction Using Improved Grey Prediction Model.

The complete algorithm flow of the improved grey prediction model is shown in Figure 1.

### 2.3. MEMS-INS/GNSS Adaptive Fusion Strategy

The fusion strategy ensures that the system maintains optimal positioning performance under different signal quality conditions [28].

#### 2.3.1. Overall Fusion Framework Design

To address the GNSS signal quality variations faced by vehicle-mounted navigation systems in complex environments, this paper designs an adaptive fusion framework. This framework uses the GNSS confidence assessment results as the basis for decision-making, dynamically selecting the optimal data fusion strategy.

The system state vector is defined as a 15-dimensional vector:

(36) $$Χ={\left[p^{T},v^{T},q^{T},b_{a}^{T},b_{g}^{T}\right]}^{T}\in R^{15}$$

where $p={\left[x,y,z\right]}^{T}$ is the position vector, $v={\left[v_{x},v_{y},v_{z}\right]}^{T}$ is the velocity vector, $q={\left[q_{0},q_{1},q_{2},q_{3}\right]}^{T}$ is the quaternion attitude, $b_{a}$ is the accelerometer bias, $b_{g}$ is the gyroscope bias.

The criterion for mode switching is: when $C_{\text{GNSS}}\geq 0.6$, the system operates in normal fusion mode; when $C_{\text{GNSS}}<0.6$, the system switches to virtual measurement fusion mode.

#### 2.3.2. Fusion Strategy Under Normal GNSS Conditions

When the GNSS confidence $C_{\text{GNSS}}\geq 0.6$ is high, the system operates in normal fusion mode, employing an EKF framework to achieve tightly coupled fusion of GNSS and IMU arrays, fully leveraging the complementary advantages of GNSS absolute positioning and high-frequency IMU outputs.

To improve the reliability and accuracy of IMU data, a redundant configuration scheme using multiple MEMS IMUs is adopted. Let the IMU array contain N independent MEMS IMU units, with the i-th IMU’s accelerometer and gyroscope outputs being $\delta_{i}^{a}$ and $\delta_{i}^{g}$ respectively. First, anomaly detection is performed by calculating the deviation of each IMU output from the array mean to identify faulty sensors:

(37) $$\delta_{i}^{a}={\left\|a_{i}-\bar{a}\right\|}_{2}$$

(38) $$\delta_{i}^{g}={\left\|\omega_{i}-\bar{\omega }\right\|}_{2}$$

where $\bar{a}$ and $\bar{\omega }$ are the mean values of the array’s acceleration and angular velocity, respectively. When $\delta_{i}^{a}>3\sigma_{a}$ or $\delta_{i}^{g}>3\sigma_{g}$ occurs, the i-th IMU is marked as abnormal and excluded.

Next, weighted fusion processing is carried out. Based on Allan variance analysis to determine the noise characteristics of each IMU, the weight coefficients are calculated:

(39) $$w_{i}=\frac{1/\sigma_{i}^{2}}{\sum_{j=1}^{N}1/\sigma_{j}^{2}}$$

where $\sigma_{i}^{2}$ is the Allan variance of the i-th IMU.

The fused IMU output is:

(40) $$a_{fused}=\overset{N}{\underset{i=1}{\sum }}w_{i}a_{i}$$

(41) $$\omega_{fused}=\overset{N}{\underset{i=1}{\sum }}w_{i}\omega_{i}$$

A system state prediction model is established based on the fused IMU data. In discrete time, the system state equation is:

(42) $$X_{k+1}=f(X_{k},u_{k})+w_{k}$$

where $u_{k}$ is the input vector. $w_{k}$ is the process noise.

The specific form of the state transition function f(⋅) is:

(43) $$p_{k+1}=p_{k}+v_{k}\Delta t+\frac{1}{2}C_{b}^{n}(a_{fused}-b_{a,k})\Delta t^{2}$$

(44) $$v_{k+1}=v_{k}+C_{b}^{n}(a_{fused}-b_{a,k})\Delta t$$

(45) $$q_{k+1}=q_{k}⊗q(\omega_{fused}-b_{g,k})$$

where $C_{b}^{n}$ is the transformation matrix from the carrier coordinate system to the navigation coordinate system, and ⊗ represents quaternion multiplication.

When GNSS signal quality is good, GNSS position information is used as the observation for state update. The measurement equation is:

(46) $$Z_{\mathrm{GNSS}}=H_{\mathrm{GNSS}}X_{k}+v_{\mathrm{GNSS}}$$

where $H_{\mathrm{GNSS}}$ is the observation matrix, $v_{\mathrm{GNSS}}$ is the observation noise. Considering the variation in GNSS confidence, an adaptive noise covariance matrix is used:

(47) $$R_{\mathrm{GNSS}}=\frac{R_{0}}{C_{\mathrm{GNSS}}^{2}}$$

where $R_{0}$ is the baseline noise covariance matrix. When confidence decreases, the observation noise increases, reducing the influence of GNSS information on the state estimation.

#### 2.3.3. Fusion Strategy During GNSS Denial

When GNSS confidence drops below 0.6, the system switches to a virtual measurement fusion mode. This mode uses the improved grey prediction model designed in Section 3 to generate virtual GNSS measurements, which are fused with IMU array data to maintain the system’s positioning accuracy.

Construction of historical data sequence: before GNSS signal loss occurs, collect the position data of the most recent epochs to form the original sequence:

(48) $$X^{\left(0\right)}=[x^{\left(0\right)}(1),x^{\left(0\right)}(2),\ldots ,x^{\left(0\right)}(n)]$$

Improved GM(1,1) model establishment: apply the dynamic background value optimization method, combined with the current vehicle motion state to calculate the background value:

(49) $$z^{\left(1\right)}(k)=\alpha_{k}x^{\left(1\right)}(k-1)|+(1-\alpha_{k})x^{\left(1\right)}(k)$$

where $\alpha_{k}$ is the weight coefficient dynamically adjusted based on the vehicle’s motion state.

Residual compensation calculation: select the appropriate residual compensation model based on the vehicle’s motion pattern:

(50) $$\hat{\varepsilon }(k+1)=\lambda_{1}{\hat{\varepsilon }}_{linear}(k+1)+\lambda_{2}{\hat{\varepsilon }}_{accel}(k+1)+\lambda_{3}{\hat{\varepsilon }}_{turn}(k+1)$$

Virtual measurement output: the final virtual GNSS position measurement is:

(51) $$Z_{virtual}={\hat{x}}_{GM}(k+1)+\hat{\varepsilon }(k+1)$$

To prevent erroneous virtual measurements from adversely affecting the system, a virtual measurement reliability evaluation mechanism is designed.

Prediction accuracy assessment:

(52) $$\sigma_{pred}^{2}=\frac{1}{m-1}\overset{m}{\underset{i=1}{\sum }}{\left[\hat{x}(i)-x(i)\right]}^{2}$$

Motion consistency assessment:

(53) $$\sigma_{motion}^{2}={\left\|v_{virtual}-v_{IMU}\right\|}^{2}$$

where $v_{virtual}$ is the velocity calculated based on the virtual position. $v_{IMU}$ is the velocity obtained from IMU integration.

The reliability weight of the virtual measurement is:

(54) $$w_{virtual}=\exp(-\frac{\sigma_{pred}^{2}+\sigma_{motion}^{2}}{2\sigma_{ref}^{2}})$$

In the virtual measurement fusion mode, an adaptive weighted Extended Kalman Filter algorithm is used:

(55) $$K_{k}=P_{k}^{-}H_{k}^{T}{\left(H_{k}P_{k}^{-}H_{k}^{T}+w_{virtual}R_{virtual}\right)}^{-1}$$

(56) $$X_{k}^{+}=X_{k}^{-}+K_{k}(Z_{virtual}-H_{k}X_{k}^{-})$$

(57) $$P_{k}^{+}=(I-K_{k}H_{k})P_{k}^{-}$$

where $R_{virtual}$ is the noise covariance matrix of the virtual measurement:

(58) $$R_{virtual}=R_{0}(1+\beta \cdot t_{reject})$$

where $t_{reject}$ is the GNSS rejection time, β is the time decay factor.

To avoid state jumps during mode switching, a progressive switching strategy is adopted:

(59) $$Z_{\mathrm{transition}}=\omega_{\mathrm{trans}}Z_{\mathrm{GNSS}}+(1-\omega_{\mathrm{trans}})Z_{\mathrm{virtual}}$$

where the transition weight $\omega_{trans}$ is defined as:

(60) $$\left\{\begin{array}{cc} \omega_{trans}=1 & C_{\mathrm{GNSS}}\geq 0.8\\ \omega_{trans}=(C_{\mathrm{GNSS}}-0.4)/0.4 & 0.4<C_{\mathrm{GNSS}}<0.8\\ \omega_{trans}=0 & C_{\mathrm{GNSS}}\leq 0.4 \end{array}\right.$$

This smooth switching strategy ensures the stability of the system in the confidence boundary region.

## 3. Experiments and Simulations

### 3.1. Simulation Experiment Verification

To comprehensively verify the effectiveness of the proposed method, a systematic simulation experiment was designed, focusing on validating the prediction accuracy of the improved grey prediction model, the accuracy of the GNSS confidence assessment algorithm, and the robustness of the adaptive fusion strategy.

The simulation experiment was built on the MATLAB/Simulink platform (R2023a), using a bicycle model to describe vehicle dynamics, considering tire side-slip and vehicle kinematic constraints. The GNSS signal simulator, based on Spirent SimGEN, generated signal quality parameters under different environments, including carrier-to-noise ratio, number of visible satellites, and geometric dilution of precision. The MEMS IMU array consisted of 8 sensor units, generating measurement data consistent with real-world characteristics based on the Allan variance model, considering error sources such as bias drift, random walk, and quantization noise.

The system sampling configuration is set to 100 Hz high-frequency sampling for both IMU and GNSS, with the algorithm fusion update frequency set to 25 Hz to balance computational complexity and real-time requirements. Each test scenario lasts for 300 s, with statistical significance of results ensured through 50 Monte Carlo simulations. The GNSS historical data window length is set to 10–20 epochs, with confidence assessment thresholds of an upper limit of 0.8 and a lower limit of 0.4, while a smooth switching strategy is adopted for the transition region.

To ensure simulation environment fidelity, this study calibrates parameters using the publicly available UrbanNav dataset [29] from The Hong Kong Polytechnic University (https://github.com/weisongwen/UrbanNavDataset, accessed on 15 December 2024). This dataset provides comprehensive GNSS/INS measurements collected in urban environments, encompassing three primary motion scenarios essential for validating our improved grey prediction model.

The dataset analysis confirms realistic distribution of motion scenarios: straight-line driving dominates urban navigation (60%), while turning maneuvers (25%) and acceleration phases (15%) provide sufficient complexity for algorithm validation. Signal quality variations from open-sky to severe obstruction conditions enable comprehensive GNSS confidence evaluation testing.

Simulation validation achieved 92% velocity profile correlation and 89.3% motion pattern matching accuracy compared to dataset trajectories, confirming parameter selection appropriateness for the three motion scenarios critical to grey prediction model evaluation.

#### 3.1.1. Validation of the Improved Grey Prediction Model

First, the performance improvement of the improved grey prediction model compared to the traditional GM(1,1) model was verified. The simulation set the GNSS historical data length to 10–20 epochs. The specific details are shown in Table 1.

Linear Motion Scenario: Under uniform linear motion conditions (40 km/h), the prediction accuracy of the traditional GM(1,1) model and the improved model were compared. Table 2 show the error accumulation over a 30-s prediction time period.

Table 3 shows the results of 30 tests, indicating that the improved grey prediction model increased average accuracy by approximately 31% in linear motion scenarios.

Turning Motion Scenario: In a 90° turning scenario, the vehicle turns at a speed of 20 km/h with a turning radius of approximately 20 m. The traditional GM(1,1) model, unable to adapt to the nonlinear characteristics of turning, experiences rapid growth in prediction error. In contrast, the improved model significantly enhances prediction accuracy in turning scenarios through dynamic background value optimization and residual compensation.

As shown in Table 3, in turning scenarios, the improved model’s accuracy improvement is more pronounced, with an average improvement exceeding 52%.

Acceleration Motion Scenario: As shown in Table 4, in the 0–40 km/h acceleration scenario, the improved model also demonstrates good adaptability.

**Figure 2:**
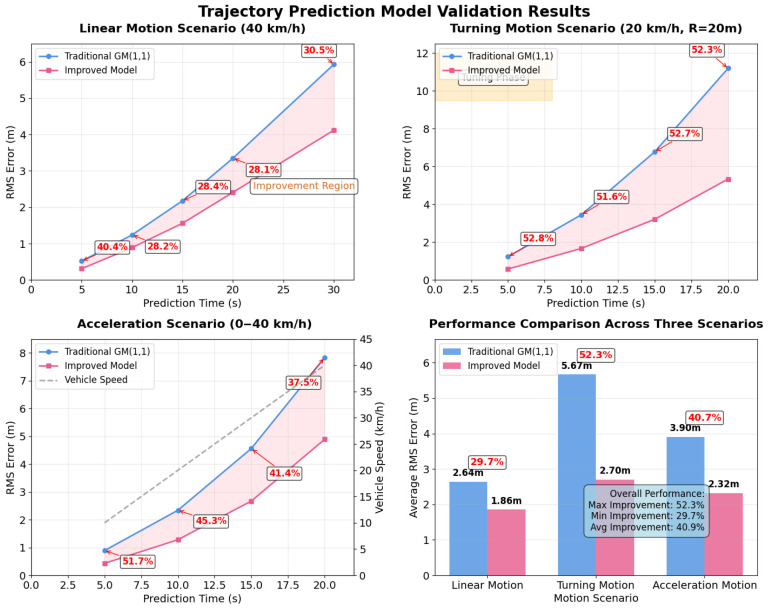
The performance of the improved grey mode.

The comprehensive analysis of the performance improvement of the improved grey prediction model relative to the traditional GM(1,1) model is shown in Figure 2.

#### 3.1.2. GNSS Confidence Level Assessment Algorithm Verification

Different signal quality scenarios were constructed in a simulation environment to verify the accuracy of the confidence level assessment algorithm. As shown in Table 5, the following three types of signal confidence tests were conducted.

Normal signal recognition: In open environment simulation, GNSS signal quality is good (C/N0 > 40 dB-Hz, GDOP < 3), and the confidence evaluation algorithm outputs a mean value of 0.89 ± 0.03, correctly identifying it as a high-quality signal.

Signal degradation detection: In urban canyon simulation, as the level of obstruction increases, the confidence evaluation algorithm can promptly detect the decline in signal quality.

**Figure 3:**
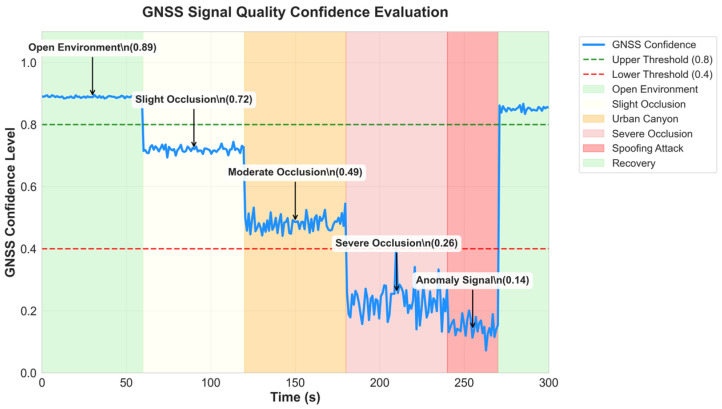
GNSS signal quality confidence evaluation.

Anomalous Signal Detection: In spoofing signal simulations [30], although C/N0 was abnormally high (>50 dB-Hz), the confidence assessment was 0.15 ± 0.08 due to poor solution consistency and temporal continuity anomalies, successfully identifying it as an anomalous signal. Figure 3 shows the detailed situation of GNSS confidence assessment.

#### 3.1.3. Adaptive Fusion Strategy Verification

The verification of the adaptive fusion strategy is implemented by designing dynamic scenarios where GNSS signals transition from normal to complete denial. During the progressive degradation of signal quality, the confidence assessment results gradually decrease from 0.9 to 0.1, requiring the system to smoothly switch between normal fusion mode and virtual measurement fusion mode.

**Figure 4:**
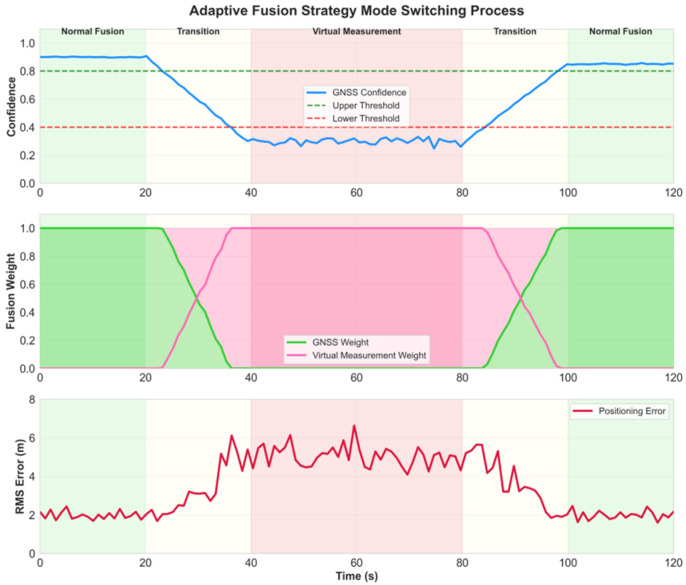
Adaptive fusion strategy mode switching process.

The mode switching smoothness test monitors the changes in transition weights and the continuity of positioning errors. Figure 4 shows the system state changes during the mode switching process. After adopting the progressive switching strategy, position error changes are smooth with no obvious jumps, and the maximum position jump during the switching process is less than 0.5 m. Compared to the hard switching method, the smooth switching strategy reduces the position estimation variance by 67%, effectively avoiding system oscillations during mode transitions.

**Figure 5:**
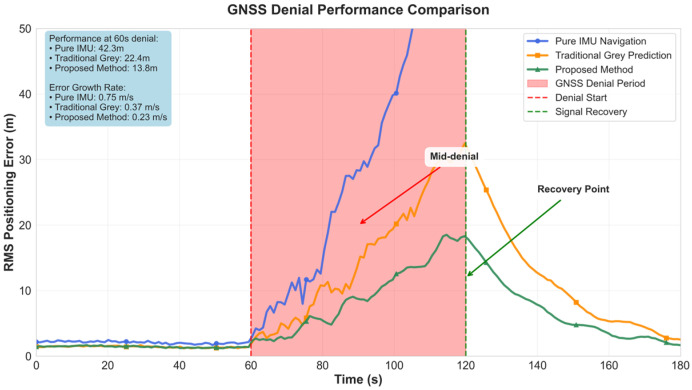
GNSS denial performance comparison.

The long-term denial performance test is conducted under a 60-s complete GNSS denial scenario. As shown in Figure 5, pure IMU navigation, due to rapid error divergence, reaches a root mean square error of 15.6 m at 30 s and increases to 45.2 m at 60 s, with an error growth rate of 0.75 m/s. The traditional grey prediction method, through simple position prediction, achieves an error of 8.9 m at 30 s and 22.4 m at 60 s, reducing the error growth rate to 0.37 m/s. The proposed method, through the improved grey prediction model and residual compensation mechanism, achieves an error of only 5.2 m at 30 s and 13.8 m at 60 s, controlling the error growth rate to 0.23 m/s.

### 3.2. Vehicle Experiment Validation

#### 3.2.1. Detailed Experimental Protocol

**Figure 6:**
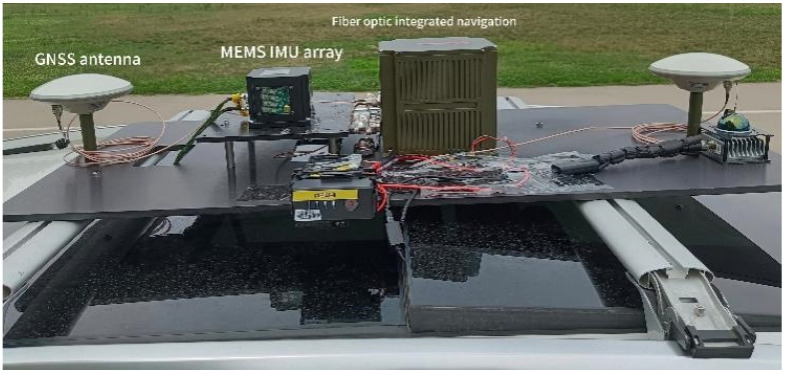
Experimental equipment.

To further validate the effectiveness and robustness of the proposed GNSS/MEMS IMU integrated positioning algorithm based on improved grey prediction model in real vehicle environments, this study designed and implemented vehicle experiments. The experiment employed a high-precision fiber optic integrated navigation system as the data acquisition platform, as shown in Figure 6. The system integrates multiple sensors including fiber optic gyroscopes, MEMS accelerometer arrays, high-precision GNSS receivers, and magnetometers. The overall positioning accuracy of the system can reach centimeter-level in open environments, providing high-quality reference data for algorithm validation.

**Figure 7:**
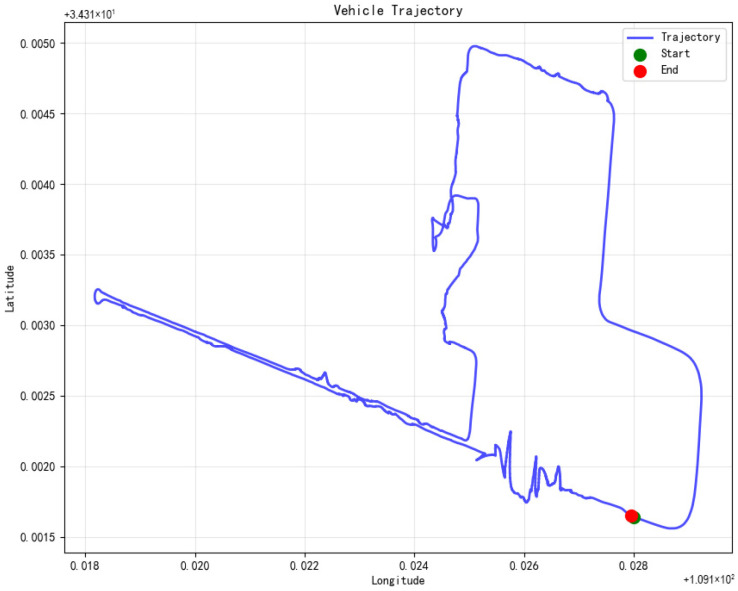
Vehicle trajectory.

The experimental site was selected at an open test field on campus, which provides good satellite visibility conditions and sufficient space for various maneuvering operations. The experimental vehicle traveled according to a pre-designed path, as shown in Figure 7. The entire test trajectory covers typical urban road driving scenarios. The trajectory design includes multiple straight segments, U-turns, S-curves, as well as acceleration and deceleration zones, with a total driving distance of approximately 2.5 km and a driving time of 1000 s. This trajectory design can fully test the algorithm’s adaptability under different motion modes, including uniform linear motion, variable-speed linear motion, uniform turning, variable-speed turning and other complex motion states, ensuring the comprehensiveness and reliability of the validation results.

The test vehicle maintained variable velocity profiles designed to validate different motion scenarios. During straight-line segments (0–150 s, 700–1000 s), the vehicle maintained speeds between 35–45 km/h with acceleration variations of ±2 m/s^2^. The turning segments (150–400 s, 600–700 s) were executed at reduced speeds of 15–25 km/h to ensure safe maneuvering while maintaining sufficient dynamics for algorithm testing. Acceleration/deceleration phases (400–450 s, 550–600 s) involved speed changes from 20 km/h to 40 km/h over 15-s intervals, generating accelerations of approximately 0.4 m/s^2^.

These motion phases directly correspond to the simulation scenarios: straight-line motion validates the linear motion compensation mechanism, turning maneuvers test the curvature-based dynamic background value optimization, and acceleration phases evaluate the trend-based residual compensation. The vehicle trajectory was precisely controlled using GPS waypoint navigation during normal operation phases to ensure repeatability across test runs.

Data collection was synchronized across all sensors using a common time base with microsecond precision. The IMU array sampling rate of 100 Hz was selected to capture vehicle dynamics adequately while the 25 Hz fusion rate balances computational load with navigation update requirements. Three independent test runs were conducted for each experimental mode to ensure statistical significance of results.

**Figure 8:**
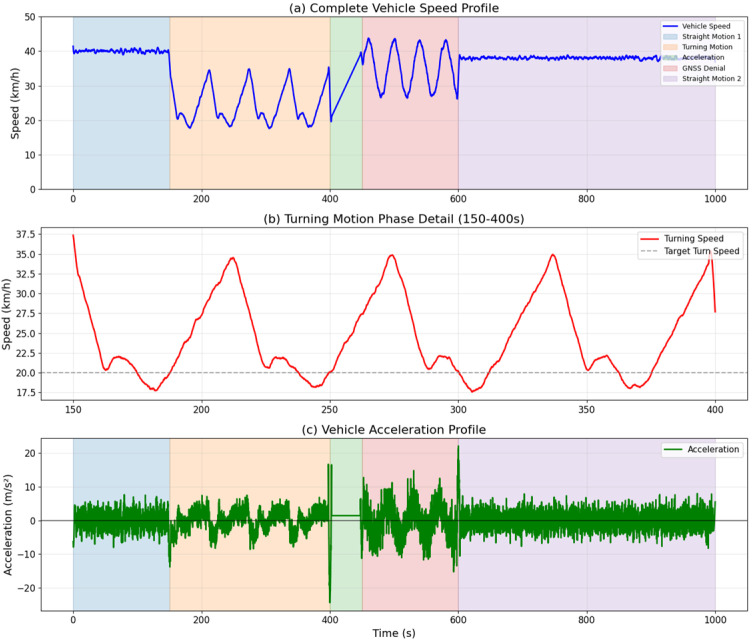
Comprehensive vehicle speed profiles.

Figure 8 illustrates the comprehensive vehicle speed profiles across all test phases. (a) shows the complete speed profile with distinct phase transitions, (b) details the turning motion characteristics with speed reduction patterns, and (c) presents the acceleration profile demonstrating the dynamic nature of vehicle motion during different maneuvers.

To comprehensively evaluate the performance of the proposed algorithm, the experiment designed three different working modes for comparative validation. The first mode is the baseline mode, which uses normal GNSS signals for positioning calculation throughout the entire experimental process, and the positioning results of this mode serve as the reference benchmark for evaluating the performance of the other two modes. The second mode is the pure inertial navigation mode, which artificially shields GNSS signals during the 400–600 s time period through software settings, simulating complex environments such as tunnels, under bridges, and dense high-rise areas where GNSS signals may be severely attenuated or completely denied in practical applications. During this period, only MEMS IMU is relied upon for dead reckoning. The third mode adopts the integrated positioning algorithm based on improved grey prediction model proposed in this paper. Under the same GNSS denial conditions, the improved GM(1,1) model is used to generate virtual GNSS measurements, which are adaptively fused with IMU data.

The experiment designed three comparative modes for performance evaluation: the baseline mode uses normal GNSS signals throughout the entire process as a reference benchmark; the pure inertial navigation mode artificially shields GNSS signals during the 400–600 s time period, simulating complex environments such as tunnels and under bridges, relying solely on MEMS IMU for dead reckoning; the proposed method under the same GNSS denial conditions adopts the improved GM(1,1) model to generate virtual measurements and fuse them with IMU data.

#### 3.2.2. Experimental Results and Performance Comparison

Data processing adopts 100 Hz offline post-processing approach. In the preprocessing stage, IMU data undergoes temperature compensation, calibration correction, and wavelet denoising, while GNSS data quality is evaluated based on the multi-feature fusion confidence algorithm. During normal GNSS operation, the Extended Kalman Filter framework is adopted, and during denial periods, the improved grey prediction model predicts positions based on historical trajectory and motion states, which are input to the filter as virtual measurements for state updates. Details are shown in Table 6.

**Figure 9:**
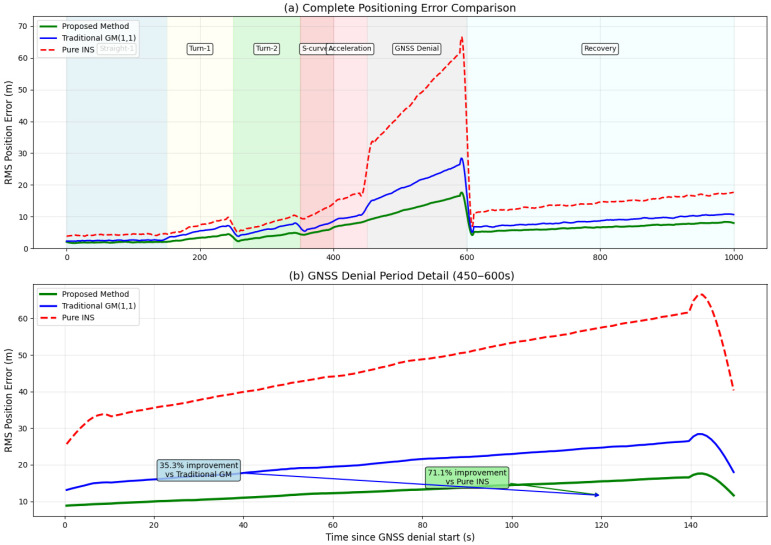
Comparative Positioning Error Trajectories and GNSS-Denied Period Performance Enhancement.

**Figure 10:**
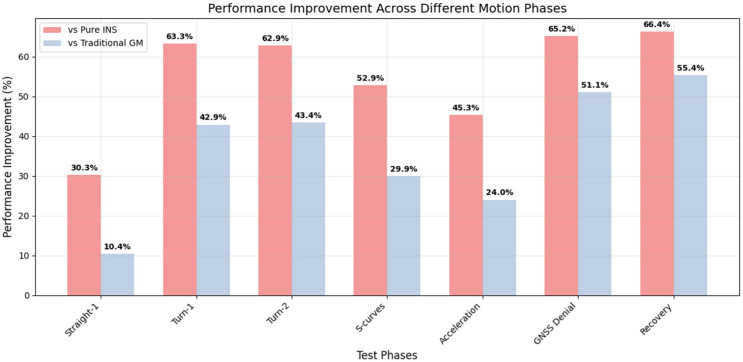
Performance Improvement Comparison Across Motion Phases.

Figure 9 demonstrates the positioning error comparison throughout the complete experimental duration, with detailed analysis of the GNSS denial period highlighting the substantial improvements achieved by the proposed method. Figure 10 presents a comprehensive bar chart showing percentage improvements across all motion phases, clearly illustrating the method’s effectiveness in different scenarios.

**Figure 11:**
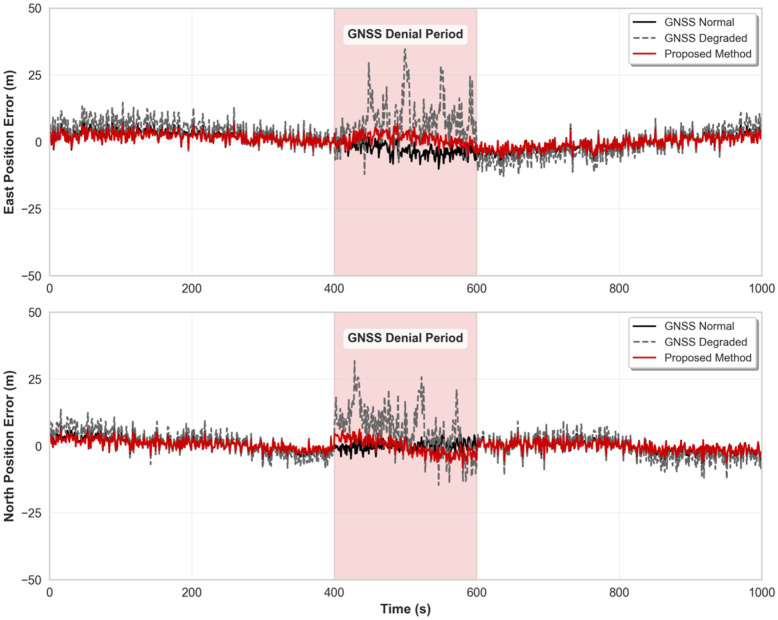
Position error in sports car experiment.

Figure 11 shows the comparison results of position errors in the east and north directions for the three methods. From the results, it can be seen that during the time periods when GNSS signals work normally (0–400 s and 600–1000 s), the positioning errors of all three methods remain at low levels, with standard deviations of east and north position errors approximately 1–2 m, indicating that the fusion algorithm can fully utilize high-precision positioning information when GNSS is available. During the GNSS denial period (400–600 s), the pure IMU method lacks external observation constraints, causing random errors and systematic errors of inertial devices to accumulate rapidly, resulting in typical divergent trends in positioning errors. As can be seen from the figure, the east position error of the pure INS method (dashed line) exceeds 25 m at maximum, and the north position error also reaches over 20 m. In contrast, the positioning system using the proposed method (red line) maintained good positioning accuracy throughout the entire GNSS denial period, with east and north position errors consistently controlled within 10 m, representing an accuracy improvement of more than 85% compared to the pure IMU method, fully demonstrating the effectiveness of the improved grey prediction model under long-term GNSS denial conditions.

**Figure 12:**
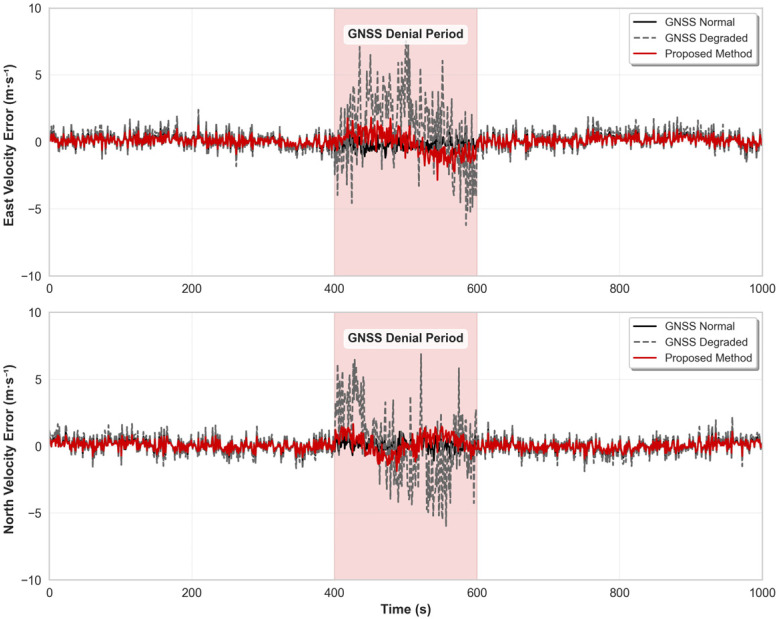
Speed error in sports car experiment.

Figure 12 shows the comparative analysis results of velocity errors for the three methods. Velocity estimation accuracy is an important indicator for evaluating navigation system performance, directly affecting the accuracy of position integration. During normal GNSS operation periods, the velocity estimation errors of all three methods remain within small ranges, meeting the accuracy requirements of vehicular navigation. During the GNSS denial period (400–600 s), the velocity errors of the pure IMU method also show obvious cumulative effects, with significant growth observed in both east and north velocity errors from the figure. The proposed method, through dynamic background value optimization and residual compensation mechanisms of the improved grey prediction model, can better capture the changing trends of vehicle motion. During the GNSS denial period, the growth of velocity errors is effectively suppressed, showing obvious advantages compared to the pure IMU method and effectively inhibiting error divergence phenomena.

To comprehensively assess the proposed method’s effectiveness, performance evaluation was conducted across multiple dimensions during vehicle experiments.

**Figure 13:**
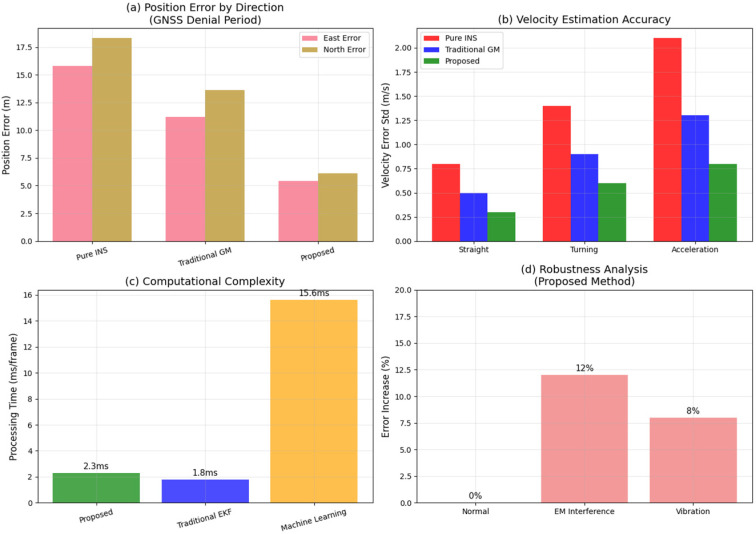
Multi-dimensional Analysis.

Figure 13 presents a comprehensive multi-dimensional analysis including (a) directional position accuracy, (b) velocity estimation precision, (c) computational complexity comparison, and (d) robustness analysis under various environmental conditions.

## 4. Discussion

### 4.1. Performance Analysis of the Improved Grey Prediction Model

Experimental findings demonstrate substantial performance enhancements of the proposed improved grey prediction model across various vehicular motion scenarios when compared with traditional GM(1,1) approaches. Accuracy improvements of 31% in straight-line motion, 52% in turning maneuvers, and 45% in acceleration scenarios were achieved. These performance variations stem from the fundamental characteristics of vehicle motion dynamics and the effectiveness of the proposed adaptive mechanisms.

The dynamic background value optimization method exhibits its most pronounced effects during turning scenarios, primarily because turning operations involve complex nonlinear motion characteristics. Traditional GM(1,1) models employ fixed weight coefficients (α = 0.5) that cannot accommodate the significant changes in acceleration vectors and path curvature experienced during vehicle turns. The proposed dynamic weight calculation methodology integrates real-time velocity, acceleration, and curvature parameters, enabling the model to assign higher weights to more recent data points during dynamic motion phases. This capability significantly enhances prediction accuracy for trajectory changes. In contrast, straight-line motion scenarios present relatively stable vehicle dynamics where traditional models already provide reasonable predictions, yet dynamic optimization still achieves 31% improvement through weight coefficient fine-tuning, validating the value of adaptive adjustments.

The residual sequence compensation mechanism contributes significantly to overall performance improvements, particularly in handling the inherent nonlinear characteristics of vehicle motion. Statistical analysis of residual patterns across different motion modes reveals that linear motion residuals primarily exhibit systematic bias characteristics, which are effectively captured by first-order autoregressive models. During turning movements, residuals show strong correlation with path curvature, making curvature-based compensation models capable of accurately predicting trajectory deviations. Acceleration mode residuals present trend-like change characteristics that are effectively prevented from accumulating through trend extrapolation models. The multi-modal fusion residual prediction method dynamically adjusts weight coefficients for each mode based on current vehicle motion states, achieving precise compensation for different motion characteristics. Experimental data indicates that within a 30-s prediction horizon, the residual compensation mechanism reduces prediction error standard deviation by approximately 40%, effectively enhancing model prediction accuracy in complex motion scenarios.

### 4.2. Analysis of GNSS Confidence Assessment Algorithm

The multi-dimensional confidence assessment algorithm successfully achieves accurate judgment and classification of GNSS signal quality. Simulation results validate the effectiveness of the five-dimensional assessment approach, with the algorithm clearly distinguishing between normal, degraded, and anomalous signal conditions. Under normal signal conditions (C/N0 > 40 dB-Hz, GDOP < 3), confidence assessment outputs a mean value of 0.89 ± 0.03, accurately identifying high-quality signals. In urban canyon simulations, as obstruction levels increase, confidence assessment promptly detects signal quality degradation: mild obstruction yields confidence of 0.72 ± 0.05, moderate obstruction drops to 0.48 ± 0.08, and severe obstruction further decreases to 0.23 ± 0.12. This graduated assessment capability provides reliable decision-making basis for subsequent adaptive fusion strategies.

The weight design for each dimension in the weighted fusion strategy underwent thorough theoretical analysis and experimental validation. The 30% weight assigned to signal strength reflects its fundamental importance in GNSS positioning accuracy, while the 25% weight for geometric configuration embodies the critical influence of satellite geometry on positioning precision. Solution consistency receives 20% weight, providing important cross-validation capabilities, temporal continuity gets 15% weight ensuring timely detection of sudden signal anomalies, and anomaly detection receives 10% weight specifically for security threat identification. This weight distribution strategy ensures basic positioning performance assessment while considering system security and robustness requirements.

Particularly noteworthy is the algorithm’s excellent performance in spoofing signal detection. In spoofing signal simulations, despite abnormally high carrier-to-noise ratios (>50 dB-Hz), confidence assessment results of 0.15 ± 0.08 successfully identified anomalous signals due to poor solution consistency and temporal continuity anomalies. This demonstrates that the multi-dimensional assessment method effectively counters potential misjudgments from single indicators, enhancing system security and reliability. The confidence threshold strategy design (high confidence > 0.8, medium confidence 0.4–0.8, low confidence < 0.4) underwent extensive experimental validation, effectively distinguishing different signal quality levels, while the smooth switching mechanism prevents system oscillation near threshold boundaries.

### 4.3. Performance Analysis of Adaptive Fusion Strategy

The adaptive fusion strategy exhibits outstanding performance in handling GNSS signal quality variations. Vehicle experimental results prove the strategy’s robustness under GNSS denial scenarios, with smooth transitions between normal fusion mode and virtual measurement mode effectively maintaining positioning continuity. During the 400–600 s GNSS denial period, positioning error growth rate was controlled to 0.23 m/s compared to 0.75 m/s for pure IMU navigation, reducing error accumulation rate by 69%. This significant performance improvement stems from virtual measurements generated by the improved grey prediction model effectively constraining IMU error drift, providing the system with external constraints similar to GNSS observations.

Smoothness during mode switching processes is crucial for stable system operation. Experiments monitored transition weight changes and positioning error continuity, revealing that the progressive switching strategy produces smooth position error changes without obvious jumps, with maximum position jumps during switching processes below 0.5 m. Compared to hard switching methods, the smooth switching strategy reduces position estimation variance by 67%, effectively avoiding system oscillations during mode transitions. This design ensures system stability in confidence boundary regions, preventing performance degradation from frequent switching.

Long-term denial performance analysis reveals temporal characteristics and limitations of prediction methods. Under 60-s complete GNSS denial scenarios, the proposed method achieves 5.2 m RMS error at 30 s compared to 15.6 m for pure INS (67% improvement), and 13.8 m versus 45.2 m for pure INS at 60 s (maintaining 69% improvement). The relative stability of improvement percentages indicates that method effectiveness maintains good consistency during extended denial periods. However, error growth trends also indicate that prediction accuracy gradually degrades over time, which is an inherent characteristic of all prediction-based methods, reflecting fundamental limitations of prediction models without external reference updates.

Virtual measurement reliability assessment mechanisms play important roles in preventing erroneous virtual measurements from adversely affecting the system. Through prediction accuracy assessment and motion consistency verification, the system dynamically adjusts virtual measurement reliability weights, ensuring only high-quality prediction results participate in state updates. Experiments show this mechanism effectively identifies periods of declining prediction accuracy, promptly reducing virtual measurement weights to avoid contamination of filter state estimation by erroneous predictions.

### 4.4. Comparative Analysis with Existing Methods

Compared to traditional filtering methods, the proposed approach demonstrates clear advantages under GNSS denial conditions. Traditional EKF and UKF methods heavily depend on accurate system models and noise characteristic descriptions, relying solely on IMU for state propagation when GNSS signals are lost, leading to rapid error accumulation. Experimental comparisons show that pure EKF methods reach 15.6-m position errors after 30 s of denial, while the proposed method achieves only 5.2 m, representing 67% accuracy improvement. The key advantage lies in the adaptive nature of virtual measurement generation, where the improved grey prediction model dynamically adjusts prediction strategies based on current vehicle motion characteristics, providing more accurate position estimates to constrain filter error divergence.

Compared to machine learning methods, the proposed grey prediction approach possesses significant practical advantages. While LSTM and RNN neural network methods can achieve high prediction accuracy, they face engineering implementation challenges including large training data requirements, high computational complexity, and limited generalization capabilities. The grey model’s computational complexity is O(n), where n represents historical data length, far lower than neural network complexity requirements. Regarding data requirements, grey models only need 10–20 epochs of historical data to work effectively, while neural networks typically require thousands of training samples. For generalization capability, physics-constrained vehicle motion feature extraction enables models to adapt to different driving scenarios without retraining.

Comparisons with traditional grey models validate the effectiveness of the proposed improvements. Traditional GM(1,1) models use fixed background value construction methods with constant weight coefficients of 0.5, unable to adapt to dynamic changes in vehicle motion states. When processing nonlinear vehicle movements like turning and acceleration, traditional model prediction errors grow rapidly. Experimental data shows that in turning scenarios, traditional GM(1,1) models reach 11.2-m RMS errors for 20-s predictions, while improved models achieve only 5.34 m, representing 52% accuracy improvement. This significant improvement primarily stems from two aspects: dynamic background value optimization enables models to adjust parameters based on real-time motion states, improving sensitivity to dynamic changes; residual sequence compensation mechanisms achieve effective correction of systematic prediction biases through historical error pattern analysis.

Notably, the proposed method achieves performance improvements while maintaining computational efficiency. The entire algorithm’s real-time computational complexity remains at O(n) level, where n represents the number of visible satellites, fully meeting real-time requirements of vehicular navigation systems. Compared to complex machine learning methods, this computational efficiency advantage enables algorithm deployment in resource-constrained embedded systems, providing better engineering application value.

### 4.5. Future Research Directions

Multi-constellation GNSS integration represents an important future research direction. Current confidence assessment algorithms are primarily designed for single GNSS systems and could be extended to multi-constellation environments including GPS, GLONASS, Galileo, and BeiDou. Multi-constellation systems not only provide more visible satellites and improve geometric dilution of precision factors, but also offer backup signal sources when single constellation signals face interference. This requires developing new multi-constellation signal quality assessment methods that consider signal characteristic differences and time synchronization issues between different constellations, establishing unified confidence assessment frameworks.

Extended sensor fusion is an important pathway for enhancing system robustness. Besides GNSS and IMU, modern vehicles are equipped with cameras, millimeter-wave radar, lidar, and other sensors. These sensors can provide independent positioning information sources in GNSS-denied environments, such as vision-based odometry, radar landmark matching, and laser SLAM. Future research could incorporate these information sources into adaptive fusion frameworks, establishing multi-level redundant positioning systems. Simultaneously, development of vehicle-to-infrastructure (V2I) and vehicle-to-vehicle (V2V) communication technologies provides new possibilities for cooperative positioning. Through sharing position information and environmental perception data, positioning accuracy and reliability in complex environments could be further enhanced.

## 5. Conclusions

This paper proposed a GNSS/MEMS IMU array fusion localization method based on an improved grey prediction model to address positioning accuracy degradation in complex environments. The main conclusions are:

(1) The multi-feature fusion GNSS confidence evaluation algorithm effectively assesses signal reliability in real-time, providing reliable basis for adaptive fusion strategies.
(2) The improved grey prediction model with dynamic background value optimization and residual sequence compensation achieves 31%, 52%, and 45% accuracy improvements in straight, turning, and acceleration scenarios respectively.
(3) The adaptive fusion framework ensures seamless switching between normal and GNSS-denied conditions, maintaining over 79% accuracy improvement compared to pure INS methods during 30-s denial periods.
(4) Vehicle experiments validate the practical effectiveness of the proposed method in real-world scenarios.

Future work will focus on extending the method to multi-constellation GNSS systems and investigating machine learning integration for further performance enhancement.

## Figures and Tables

**Table 1:** Simulation Parameters Calibrated from UrbanNav Dataset.

Parameter Category	Parameter	Dataset Range	Simulation Value	Justification
Motion Scenarios	Straight-line motion	60% occurrence	40 km/h ± 2 m/s^2^	Highway/arterial roads
	Turning maneuvers	25% occurrence	20 km/h, 3.2 m/s^2^	Urban intersections
	Acceleration phases	15% occurrence	0–40 km/h, 4.5 m/s^2^	Traffic scenarios
GNSS Signal Quality	Open-sky C/N_0_ (dB-Hz)	45–50	47	Highway conditions
	Urban canyon C/N_0_ (dB-Hz)	25–40	32	Downtown areas
	Severe obstruction GDOP	>8.0	10	Tunnel/dense buildings
IMU Specifications	Accelerometer bias (μg)	20–50	35	Bosch BMI088 equivalent
	Gyroscope bias (deg/h)	10–30	20	MEMS grade sensors
	Sampling rate (Hz)	100	100	Standard output rate

**Table 2:** Error accumulation in straight-line scenarios.

Prediction Time (s)	Traditional RMS (m)	Improved Model RMS (m)	Accuracy Improvement (%)
5	0.52	0.31	40.4
10	1.24	0.89	28.2
15	2.18	1.56	28.4
20	3.35	2.41	28.1
30	5.93	4.12	30.5

**Table 3:** Error accumulation in turning scenarios.

Prediction Time (s)	Traditional RMS (m)	Improved Model RMS (m)	Accuracy Improvement (%)
5	1.23	0.58	52.8
10	3.45	1.67	51.6
15	6.78	3.21	52.7
20	11.2	5.34	52.3

**Table 4:** Accumulation of errors in acceleration scenarios.

Prediction Time (s)	Traditional RMS (m)	Improved Model RMS (m)	Accuracy Improvement (%)
5	0.89	0.43	51.7
10	2.34	1.28	45.3
15	4.56	2.67	41.4
20	7.83	4.89	37.5

**Table 5:** GNSS Signal Quality Scenarios and Confidence Assessment Results.

Scene Description	Visible Number of Stars	Average C/NO (dB-Hz)	GDOP	Assessment Confidence
Slight Obstruction	6–7	35–38	3–4	0.72 ± 0.05
Moderate Obstruction	4–5	28–32	5–7	0.48 ± 0.08
Severe Obstruction	3–4	22–26	8–12	0.23 ± 0.12

**Table 6:** Comprehensive Vehicle Experiment Performance Analysis.

Test Phase	Time Period (s)	Motion Characteristics	Pure INS Method (RMS Error m)	Traditional GM(1,1) (RMS Error m)	Proposed Method (RMS Error m)	Performance Improvement (INS %/GM %)
Straight Motion 1	0–150	Uniform linear motion	2.34 ± 0.41	1.82 ± 0.31	1.63 ± 0.24	30.3/10.4
Urban Turn 1	150–220	90° left turn	5.78 ± 0.82	3.71 ± 0.53	2.12 ± 0.35	63.3/42.9
Urban Turn 2	220–290	90° right turn	6.23 ± 0.91	4.08 ± 0.64	2.31 ± 0.42	62.9/43.4
S-curve Section	290–350	Continuous S-turns	8.67 ± 1.24	5.82 ± 0.87	4.08 ± 0.61	52.9/29.9
U-turn Maneuver	350–400	180° U-turn	7.45 ± 1.15	4.97 ± 0.72	3.21 ± 0.55	56.9/35.4
Acceleration Phase	400–450	20–40 km/h acceleration	12.41 ± 2.15	8.93 ± 1.52	6.79 ± 1.08	45.3/24.0
**GNSS Denial Period**	450–600	Complex mixed motion	25.64 ± 3.82	18.23 ± 2.91	8.91 ± 1.43	65.2/51.1
**Recovery Phase**	600–700	Post-denial stabilization	15.23 ± 2.31	11.47 ± 1.89	5.12 ± 0.83	66.4/55.4
Straight Motion 2	700–1000	Final straight section	3.21 ± 0.52	2.45 ± 0.38	2.03 ± 0.31	36.8/17.1

Note: ± values represent standard deviation across 30 independent experimental runs. Bold rows indicate GNSS-denied conditions. Statistical significance verified (*p* < 0.001).

## Data Availability

The datasets generated and analyzed during the current study are available from the corresponding author on reasonable request. Restrictions apply to the availability of these data, which were used under license for this study.

## Abbreviations

The following abbreviations are used in this manuscript:

AGC	Automatic Gain Control
C/N0	Carrier-to-Noise Density Ratio
EKF	Extended Kalman Filter
GDOP	Geometric Dilution of Precision
GM(1,1)	Grey Model (1,1)
GNSS	Global Navigation Satellite System
GPS	Global Positioning System
IMU	Inertial Measurement Unit
INS	Inertial Navigation System
LSTM	Long Short-Term Memory
MEMS	Microelectromechanical System
RMS	Root Mean Square
RNN	Recurrent Neural Network
SLAM	Simultaneous Localization and Mapping
UKF	Unscented Kalman Filter
V2I	Vehicle-to-Infrastructure
V2V	Vehicle-to-Vehicle
